# Understanding the Nature of Face Processing in Early Autism: A Prospective Study

**DOI:** 10.1037/abn0000648

**Published:** 2022-08

**Authors:** Charlotte Tye, Giorgia Bussu, Teodora Gliga, Mayada Elsabbagh, Greg Pasco, Kristinn Johnsen, Tony Charman, Emily J. H. Jones, Jan Buitelaar, Mark H. Johnson, BASIS team

**Affiliations:** 1 Department of Child and Adolescent Psychiatry and MRC Social, Genetic and Developmental Psychiatry Centre, Institute of Psychiatry, Psychology and Neuroscience, King’s College London; 2 Department of Cognitive Neuroscience, Donders Institute for Brain, Cognition and Behavior, Radboud University Medical Center; 3 Centre for Brain and Cognitive Development, Birkbeck College, University of London; 4 Department of Psychiatry, McGill University; 5 Department of Psychology, Institute of Psychiatry, Psychology and Neuroscience, King’s College London; 6 Mentis Cura, Reykjavík, Iceland; 7 South London and Maudsley NHS Foundation Trust (SLaM), London, United Kingdom; 8 Department of Cognitive Neuroscience, Karakter Child and Adolescent Psychiatry University Centre; 9 Department of Psychology, University of Cambridge

**Keywords:** autism, ERP, face processing, machine learning, prospective longitudinal study

## Abstract

Dimensional approaches to psychopathology interrogate the core neurocognitive domains interacting at the individual level to shape diagnostic symptoms. Embedding this approach in prospective longitudinal studies could transform our understanding of the mechanisms underlying neurodevelopmental disorders. Such designs require us to move beyond traditional group comparisons and determine which domain-specific alterations apply at the level of the individual, and whether they vary across distinct phenotypic subgroups. As a proof of principle, this study examines how the domain of face processing contributes to the emergence of autism spectrum disorder (ASD). We used an event-related potentials (ERPs) task in a cohort of 8-month-old infants with (*n* = 148) and without (*n* = 68) an older sibling with ASD, and combined traditional case-control comparisons with machine-learning techniques for prediction of social traits and ASD diagnosis at 36 months, and Bayesian hierarchical clustering for stratification into subgroups. A broad profile of alterations in the time-course of neural processing of faces in infancy was predictive of later ASD, with a strong convergence in ERP features predicting social traits and diagnosis. We identified two main subgroups in ASD, defined by distinct patterns of neural responses to faces, which differed on later sensory sensitivity. Taken together, our findings suggest that individual differences between infants contribute to the diffuse pattern of alterations predictive of ASD in the first year of life. Moving from group-level comparisons to pattern recognition and stratification can help to understand and reduce heterogeneity in clinical cohorts, and improve our understanding of the mechanisms that lead to later neurodevelopmental outcomes.

## General Scientific Summary

This study suggests that neural processing of faces is diffusely atypical in autism spectrum disorder, and that it represents a strong candidate predictor of later social skills and diagnosis at an individual level in the first year of life. Furthermore, a subgroup of infants developing ASD showed reduced neural responses to faces in infancy but increased sensory sensitivity in toddlerhood.

Autism spectrum disorder (ASD) is defined on the basis of social and communication impairment, restricted patterns of behaviors and interests, and sensory anomalies in early childhood (American Psychiatric Association, 2013). ASD is characterized by high heterogeneity, expressed as considerable variability across individuals in terms of both clinical manifestations and underlying biology (E. J. Jones et al., 2014; Lai et al., 2013; Vorstman et al., 2017). Parsing this heterogeneity is a main theme of theoretical initiatives in mental health research, such as the Research Domain Criteria (RDoC) Framework (Insel et al., 2010). Theoretical models like RDoC propose a shift from unitary diagnostic labels toward determining how underlying impairments in a set of core domains contribute to diagnostic phenotypes. This may facilitate greater individualization of treatment options by enabling individuals to be characterized by dimensional scores reflecting domain functioning, hypothetically facilitating neurobiologically informed treatments. Studying RDoC domains in early development, prior to the onset of behavioral symptoms, might be particularly critical for understanding how alterations in these domains contribute to symptom emergence. To do this, it is fundamental to adopt analytic strategies that profile a selected domain at the individual level, and understand its developmental link to ASD.

While most genetic studies treat ASD as a unitary clinical category, the majority of genetic liability to ASD is thought to be spread across many genes with individually small and pleiotropic effects (Huguet et al., 2016). A recently proposed framework suggests that these genetic factors act through the critical aggregation of earlier-interacting liabilities in contributing to the later clinical expression of ASD (Constantino, 2018). These liabilities are best described as endophenotypes, quantitative heritable neuropsychiatric alterations that can be identified in the general population as continuously distributed traits (Bearden & Freimer, 2006). A leading candidate domain in the mechanisms underlying ASD development is social cognition, and more specifically, face processing (Dawson et al., 2012; Dawson et al., 2005).

From the first year of life, infants with later ASD demonstrate emerging atypicalities in social-communicative behavior, such as a declining interest in human faces (W. Jones & Klin, 2013; Maestro et al., 2002; Osterling & Dawson, 1994). These behavioral changes appear to be accompanied by atypical neural responses to faces as measured by event-related potentials (ERPs), which provide the resolution required to investigate different temporal stages of information processing and can be obtained at younger ages than behavioral assessments (de Haan, 2007). The early sensory P1, and later cortical N290 and P400 responses, have been shown to be consistently modulated in face processing tasks in the first year of life, and the N290/P400 are argued to be developmental precursors of the well-established adult N170 response, reflecting processing of semantic and structural aspects of faces (de Haan et al., 2003). While some studies have suggested that low-level sensory responses to faces in infancy, indexed by the P1 ERP, is associated with better social development (E. Jones, Dawson, & Webb, 2018), atypicalities in higher-level cortical processing of faces and gaze have been reported in toddlers and children with ASD (Dawson et al., 2002; Grice et al., 2005; Webb et al., 2011). From 6 months of age, altered later cortical responses to faces versus nonfaces, and reduced differentiation of faces that shift gaze toward versus away from the viewer, are observed in infants with later ASD, as indexed by the P400 ERP (Elsabbagh et al., 2012; E. Jones et al., 2016). In “social first” theories, atypicalities in social engagement and information processing mutually amplify each other over developmental time, reducing opportunities for social learning and contributing to the atypical development of social communication that is characteristic of ASD (Elsabbagh, 2020). Thus, neural responses to faces represents one of the early measurable liabilities that could be aggregated with other factors to ultimately lead to ASD. However, little is known about how and what alterations in face processing contribute to ASD emergence.

While previous research converges in identifying face processing as a relevant domain in ASD, studies report greater divergence in the nature of ASD-associated alterations. Neural responses to faces can be characterized by many different features of an averaged waveform, which are hypothesized to reflect different underlying cognitive processes. Although the general conclusion is one of altered face processing, this diversity could reflect (a) consistent differences in lots of different specific aspects of face processing that are masked in different studies by their theory-driven focus on one or two components; (b) individually specific profiles of specific alterations that will or will not be apparent at the group level depending on its composition; and/or (c) distinct profiles of alterations in coherent subgroups of individuals developing ASD, again apparent or not at the group level depending on the sample composition. To understand these patterns, it is important to couple top-down and data-driven analytic strategies with larger samples and individual-level data analysis.

Here, we used three analytic strategies to ask how atypicalities in the domain of face processing contribute to later ASD. First, we used a prospective approach because it enables the investigation of causal mechanisms. Based on a sibling recurrence rate of around 20% (Ozonoff et al., 2011), prospective studies of infants with an older sibling with ASD (“infant siblings”) represent a powerful research design to identify precursors of symptom emergence in ASD (E. J. H. Jones et al., 2019). Second, we combined group-based comparisons with investigating individual effects through data-driven multifeature machine learning approaches for prediction of traits and diagnosis. Since domains are multifaceted rather than single cognitive processes, this approach enables an examination of the consistency of results across both top-down prediction and bottom-up discovery and construction of a robust multivariate model for data integration and prediction of outcome at an individual level (Arbabshirani et al., 2017; Rosenberg et al., 2018; Yahata et al., 2017). Third, we decomposed heterogeneity among infants at elevated likelihood for ASD with a clustering approach based on the domain under investigation (here social cognition/face processing) to examine whether we could identify subgroups with qualitatively different face processing alterations (Lombardo et al., 2016; Zhao & Castellanos, 2016). Moving from group-level comparisons to pattern recognition and stratification, this study promotes the use of novel, individual-level approaches for a dimensional understanding of brain development.

## Methods and Materials

### Participants

This study included 247 infants at elevated likelihood (EL), based on having an older biological sibling with ASD, and at typical likelihood (TL) of developing ASD, recruited from the British Autism Study of Infant Siblings (www.basisnetwork.org) across two independent cohorts. Specifically, 54 EL (21 male) and 50 TL infants (21 male) participated in cohort 1 (Elsabbagh et al., 2012), and 116 EL (64 male) and 27 TL (14 male) in cohort 2. TL controls were full-term infants (gestational age 38–42 weeks) recruited from a volunteer database at the Birkbeck Centre for Brain and Cognitive Development. Infants were seen for the face/gaze ERP task when they were approximately 8 months old (Table S1/S2 in the online supplemental materials). Subsequently, 237 were seen for assessment around their third birthday by an independent team. Four TL and 2 EL children were absent for the 36-month visit but were included in the analysis based on assessments at the previous visits. Among the remaining 243 infants, 27 were excluded based on quality of EEG data, resulting in a final sample of 216 infants (TL = 68, EL-no ASD = 115, EL-ASD = 33; see Table S3 for details). All procedures were in agreement with ethical approval granted by the London Central NREC (approval codes 06/MRE02/73, 08/H0718/76), and one or both parents gave informed consent to participate in the study.

### Clinical Assessment

The Autism Diagnostic Observation Schedule (ADOS)—generic (Lord et al., 2000), a semistructured observational assessment, and the Social Communication Questionnaire (SCQ; Rutter et al., 2003), a screening tool for ASD, were used to assess current symptoms of ASD at 36 months. The Autism Diagnostic Interview—Revised (ADI-R), a structured parent interview, was completed with parents of EL infants in cohort 1 and all children in cohort 2. These assessments were conducted without blindness to risk-group status by (or under the close supervision of) clinical researchers with demonstrated research-level reliability. The Mullen Scales of Early Learning (Mullen, 1995) and the Vineland Adaptive Behavior Scale (VABS; Sparrow et al., 1984) were used to measure, respectively, cognitive abilities and adaptive functioning at each visit.

Experienced researchers and the lead clinician (TC) determined the best estimate clinical outcome by reviewing all available information from visits performed. Of the 148 EL participants included in analyses, 33 (22.3%) participants met criteria for ASD (hereafter EL-ASD) and the remaining 115 (77.7%) participants did not meet criteria for ASD (hereafter EL-no ASD), using ICD-10 criteria (cohort 1) or *DSM–5* (cohort 2). There was a significant difference in clinical outcome by gender, χ^2^(2) = 13.5, *p* = .001, with more males receiving an ASD diagnosis than females (odds ratio, *OR* = 4.84; 95% confidence interval [CI: 1.93, 12.1]; *p* < .001). No TL children met criteria for ASD at the 36-month assessment and none had a community clinical diagnosis.

### Electrophysiological Measures

The task was the same as in Elsabbagh et al. (2012), designed to assess responses to the following contrasts: (1) faces (valid static (irrespective of gaze direction) vs. visual noise stimuli presented at the beginning of each block); (2) static gaze (faces with direct vs. averted gaze); and (3) dynamic gaze shifts (gaze toward vs. away from the infant; see online supplemental materials). EEG was recorded from a 128 channel Hydrocel Sensor Net. Following artifact detection and rejection (see online supplemental materials), stimulus-locked epochs (−200 to 800ms peristimulus window) were averaged for each of the three contrasts. Peak amplitude and latency of the averaged P100, N290, and P400 across occipito-temporal channels for each stimulus/contrast were used as input features for subsequent analyses.

### Statistical Analysis

#### Group-Based Comparison

**Figure 1 fig1:**
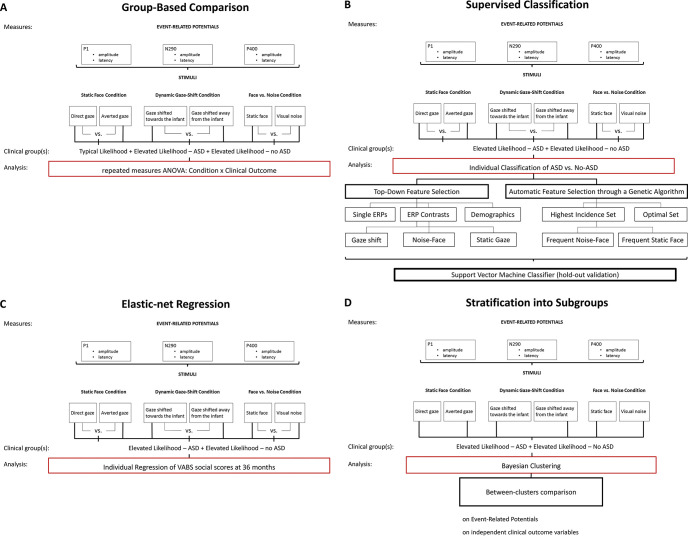
Flowchart of Statistical Analysis Strategy *Note*. This figure illustrates the different analyses performed in this study. Specifically, group-level comparison (A); supervised classification for prediction of categorical outcome at 36 months of age (B); elastic-net regression for prediction of dimensional outcome at 36 months of age (B); and stratification into subgroups of infants at elevated likelihood for autism (D). See the online article for the color version of this figure.

A repeated-measures ANOVA was conducted on each ERP parameter, with contrast as the within-subjects factor and group as the between-subjects factor (Figure 1A). A set of analyses was run with cohort as an additional between-subjects factor and followed up with post hoc *t* tests to compare ERP amplitude and latency of the EL-ASD group against other groups. Sidak correction was used to correct for multiple testing. Covariates (age at time of EEG acquisition, MSEL visual reception and fine motor (nonverbal) t-score at 36 months) were entered into a second round of analyses. Gender was not a significant covariate in any analysis and was not retained. Analyses were performed on SPSS v22 (http://www.ibm.com/analytics/us/en/technology/spss).[Fig fig1]

#### Supervised Classification

A subsample of 144 EL infants was selected based on having at least 70% of ERP data available. There were no significant differences between infants with complete and missing data in age, *t*(138) = −1.46, *p* = .15, gender, χ^2^(1) = 2.08, *p* = .15, clinical outcome, χ^2^(1) = 0.002, *p* = 1, or cognitive level at 8 months, *t*(128) = 0.07, *p* = .94, providing reasonable evidence to consider the pattern of missing data as “missing at random.” We used imputation through expectation maximization to handle missing data. The sample was split into a main sample (70% of sample, *n* = 101) for model selection and training, and a separate holdout sample (30% of sample, *n* = 43) for validation, with stratification for binary outcome (i.e., ASD vs. no-ASD). Standardized average and differential ERP responses to the different stimuli conditions were used as predictive features, together with gender and age to account for their confounding effect (see Table S1).

We performed top-down and bottom-up feature selection to extract information about the most relevant features for prediction of ASD (Dash, & Liu, 1997). For top-down feature selection, we manually selected feature sets based on stimuli and type of measure (Figure 1B). For bottom-up feature selection, we used a genetic algorithm (Back, 1996; Jóhannesson et al., 2002; Snaedal et al., 2012); online supplemental materials) based on the optimization of the area under the curve (AUC) of a 10-fold cross-validated Support Vector Machine classifier with linear kernel. AUC is an effective and combined measure of sensitivity and specificity, which allows to test the inherent ability of the predictor, providing a useful metric to evaluate diffusivity of the predictive features within the examined population (Kumar & Indrayan, 2011). Selection resulted in the feature set providing the highest AUC over repeated (*n* = 100) evolution (“optimal” set), the set of features with highest incidence (>80%, “highest incidence” set) among the best performing feature sets (AUC >85%), and condition-specific subsets of the “highest incidence set” (Figure 1B). We validated the different classifiers on the holdout sample and evaluated performance through AUC, sensitivity, specificity, accuracy, negative predictive power, and positive predictive power from the ROC curve, with bootstrapped (*n* = 10000) 95% confidence intervals (CI). We used a shuffle test with *n* = 10000 repetitions (Golland & Fischl, 2003) to test for significant differences of classification performance (AUC) from chance level prediction, and for significant differences in performance between the best performing classifier and the other classifiers. Analyses were completed using the *LIBSVM* toolbox (Chang & Lin, 2011) and custom scripts implemented on Matlab R2016b (MATLAB 9.1, The MathWorks Inc., Natick, Massachusetts, 2016).

#### Elastic-Net Regression

Regression with elastic-net regularization (Zou & Hastie, 2005) was used to select relevant ERP features for prediction of social skills in toddlerhood (VABS Socialization score at 36 months) on the same subsample of EL infants included in the classification analysis (*n* = 144), using the same ERP variables as predictors (Figure 1C). As preprocessing, skewness of predictors was checked to be lower than 0.7, and none of them needed to be transformed, while all variables were standardized. Leave-one-out cross-validation was used to cross-validate the predictive model, and nested 10-fold cross-validation with 10 repetitions was used for parameter optimization based on minimization of the root mean squared error (RMSE). To evaluate predictive performance, we computed RMSE and the relative error (RMSE/range of outcome scores). Furthermore, we computed Pearson’s correlation between predicted and observed variables, and checked residuals for normality and absence of heteroscedasticity. 95% confidence interval (CI) for RMSE was computed using bootstrap with *n* = 1000 repetitions, while the *p* value was computed through a shuffle test (Golland & Fischl, 2003).

#### Stratification Into Subgroups

To test whether neural processing of faces can define meaningful subgroups in infants at elevated likelihood for developing ASD, we used Bayesian hierarchical clustering (Marrelec et al., 2015; Savage et al., 2009) on averaged ERP responses (Figure 1D) from infants in the EL group (*n* = 144) as a model-based bottom-up process using marginal likelihoods to decide which clusters to merge. As number of clusters is determined automatically, we tested stability of results through leave-one-out cross-validation. To characterize the identified clusters, we investigated differences between clusters in face processing through an analysis of variance (ANOVA). Next, we evaluated clustering performance by examining the association of cluster membership with clinical outcome variables (ADOS, ADI-R, SCQ, MSEL, VABS) at 36 months through ANOVA (see Table S7). Holm-Bonferroni correction was used to correct significance of ANOVAs for multiple comparisons separately for ERPs and clinical outcome variables. For significant findings, we used post hoc pairwise Tukey’s tests. Analyses were implemented in *R*.

## Results

### Group-Level Differences

**Figure 2 fig2:**
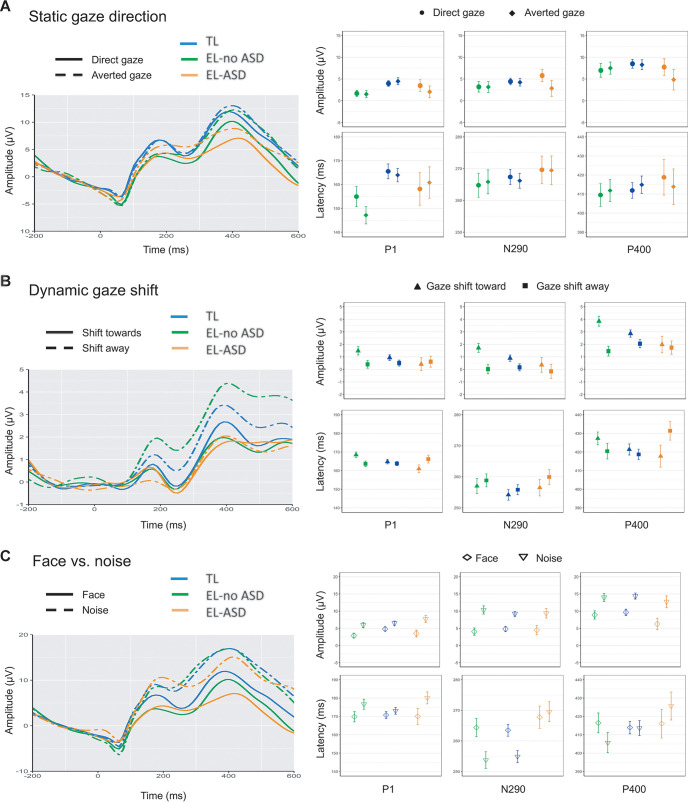
Grand Average Event-Related Potentials *Note*. Grand average ERPs across contrasts and groups over task-sensitive occipito-temporal channels (left) and means and standard errors of amplitude and latency of the three face-sensitive ERP components in each group (right). See the online article for the color version of this figure.

We found a significant Condition × Outcome interaction on N290 latency in the face–noise contrast. Specifically, the EL-ASD group did not show a stimulus differentiation, while the TL (*p* = .010, *d* = 0.61) and EL-no ASD (*p* = .021, *d* = 0.52) groups showed longer latency to faces compared to noise, with no difference between TL and EL-no ASD groups (*p* = .551, *d* = 0.10; Figure 2). This did not vary by cohort, *F*(1, 170) = 0.76, *p* = .386, and neither of the covariates had a significant interaction (*p*s > .41).[Fig fig2]

There was a significant Condition × Outcome group interaction on P1 latency, *F*(2, 213) = 4.95, *p* = .008, P400 amplitude, *F*(2, 212) = 4.13, *p* = .017, and latency, *F*(2, 208) = 3.51, *p* = .032. Specifically, the EL-ASD group had longer P1 latency to gaze shifting away versus toward, while the opposite effect was observed in the TL (*p* = .002, *d* = 0.74) and EL-no ASD groups (*p* = .047, *d* = 0.43), with no difference between TL and EL-no ASD (*p* = .122, *d* = 0.26). There was no significant interaction with cohort, *F*(2, 212) = 1.48, *p* = .226. The Condition × Outcome interaction became a trend when age and nonverbal ability were entered as covariates, *F*(2, 201) = 2.45, *p* = .089; lower nonverbal ability was associated with longer P1 latency to gaze shifting toward versus away (r = −.18, *p* = .008), with no association with age, *r* = .07, *p* = .32. Next, the EL-ASD group showed longer P400 latency to gaze shifting toward versus away from the viewer, with an opposite effect in TL (*p* = .011, *d* = 0.55) and EL-no ASD (*p* = .021, *d* = 0.47), and no significant difference between TL and EL-no ASD (*p* = .572, *d* = 0.10). This did not vary by cohort, *F*(2, 207) = 0.91, *p* = .342, and was not influenced by covariates (*p*s > .24). Post hoc *t* tests revealed significant differences between EL-ASD and TL (*p* = .009, *d* = 0.60) and EL-no ASD (*p* = .019, *d* = 0.47), but not between TL and EL-no ASD (*p* = .488, *d* = 0.12). See online supplemental materials for an additional analysis on the association between findings for P1 and P400 latency. Finally, there was enhanced P400 amplitude to gaze shifting toward versus away in the EL-ASD group (*p* = .016, *d* = 0.46) and EL-no ASD group (*p* = .014, *d* = 0.41), with no difference between EL groups (*p* = .482, *d* = 0.12) and an opposite effect in the TL group. There was no interaction with cohort, *F*(1, 211) = 0.27, *p* = .605, nor with covariates (*p*s > .19).

### Individual-Level Prediction of Diagnosis

**Figure 3 fig3:**
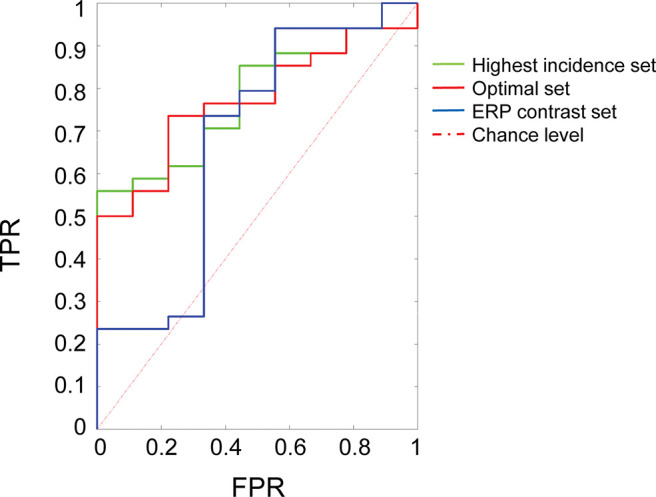
Classification Performance *Note*. Receiver operating characteristic (ROC) curve for classifiers using different set of features to classify EL-ASD among EL siblings. Random predictors result in bisecting lines as ROC curves (dashed line), while deviations in the upper hemifield indicate an increase in predictive accuracy. Only classifiers with a classification performance significantly different from chance level (assessed through a shuffle test) are included in this figure. TPR = true positive rate or sensitivity; FPR = false positive rate, or 1-specificity. See the online article for the color version of this figure.

Combined sets of brain responses including both early sensory (P1) and later higher-level ERP components (N290 and P400) in response to direct and averted gaze, static faces and visual noise (see Figure 3) provided the best predictive accuracy for ASD category at an individual level, while classification performance for condition-specific subsets of features showed largely poor predictive power. Specifically, the “Optimal” set provided the best predictive algorithm for ASD outcome at 36 months (Table 1), with an AUC of 77.1% (95% CI [61.1, 90.5], *p* = .01), significantly higher than classifiers built on condition-specific subsets selected *top-down*. Compared to the “Highest Incidence” set, which provided a nominally but not significantly higher classification accuracy (AUC = 77.5%, 95% CI [61.8, 90.2], *p* = .02; between-classifier *p* = .15), the “Optimal” set provided a better sensitivity to the ASD cases (73.5% compared to 55.9%). Of note, gender and age were selected less than 6% of the times among best performing classifiers, suggesting that prediction did not depend on these confounding variables. Table S4 shows details on classification performance.[Fig fig3][Table tbl1]

### Individual-Level Prediction of Social Traits

**Figure 4 fig4:**
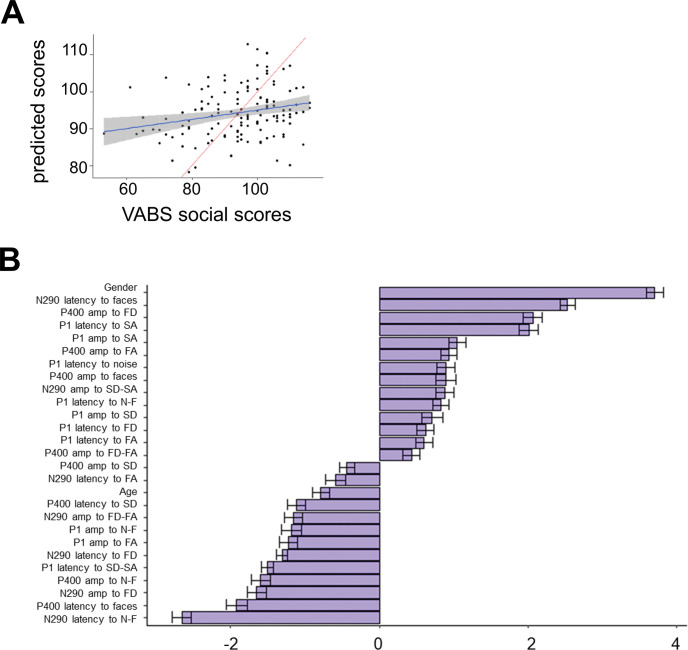
Prediction of Social Skills in Toddlerhood *Note*. This figure shows results from the elastic-net regression analysis. Specifically, panel A shows predicted against observed values of VABS socialization scores at 36 months. The experimental regression line is depicted with a shaded area for the 95% confidence interval, compared to the chance level regression line shown separately. Panel B shows average and standard deviation of coefficients always selected over cross-validation folds (*n* = 144), indicating the relevant variables for individual prediction of social skills. Coefficients are in standard units. SD = gaze shifts directed toward the infant; SA = gaze shifts directed away from the infant; FD = direct gaze; FA = averted gaze; *N* = visual noise; *F* = static face irrespective of gaze direction. See the online article for the color version of this figure.

Prediction of stronger social skills in toddlerhood using neural responses to faces and visual noise in infancy was promising (see Figure 4A), with an average RMSE = 13.17 (95% CI [11.7, 14.6]; *p* = .002) corresponding to 20.9% error relative to the score range in the sample, and a correlation between predicted and observed values β = 0.24, *t*(137) = 2.86, *p* = .005. Of the 27 features relevant to prediction (Figure 4B), N290 latency response to the face-noise contrast had the largest negative correlation to outcome, with faster N290 response to noise compared to faces associated to better social functioning in toddlerhood. There was a large overlap between predictive sets of features at the dimensional and categorical levels. Of note, gender had the largest correlation to later social skills level, indicating that females tend to have higher social scores at age 3. We also found a negative association between age of ERP assessment and level of social functioning at age 3, which likely indicates a bias in the voluntary recruitment for the study.[Fig fig4]

### Stratification Into Subgroups of EL Infants

**Figure 5 fig5:**
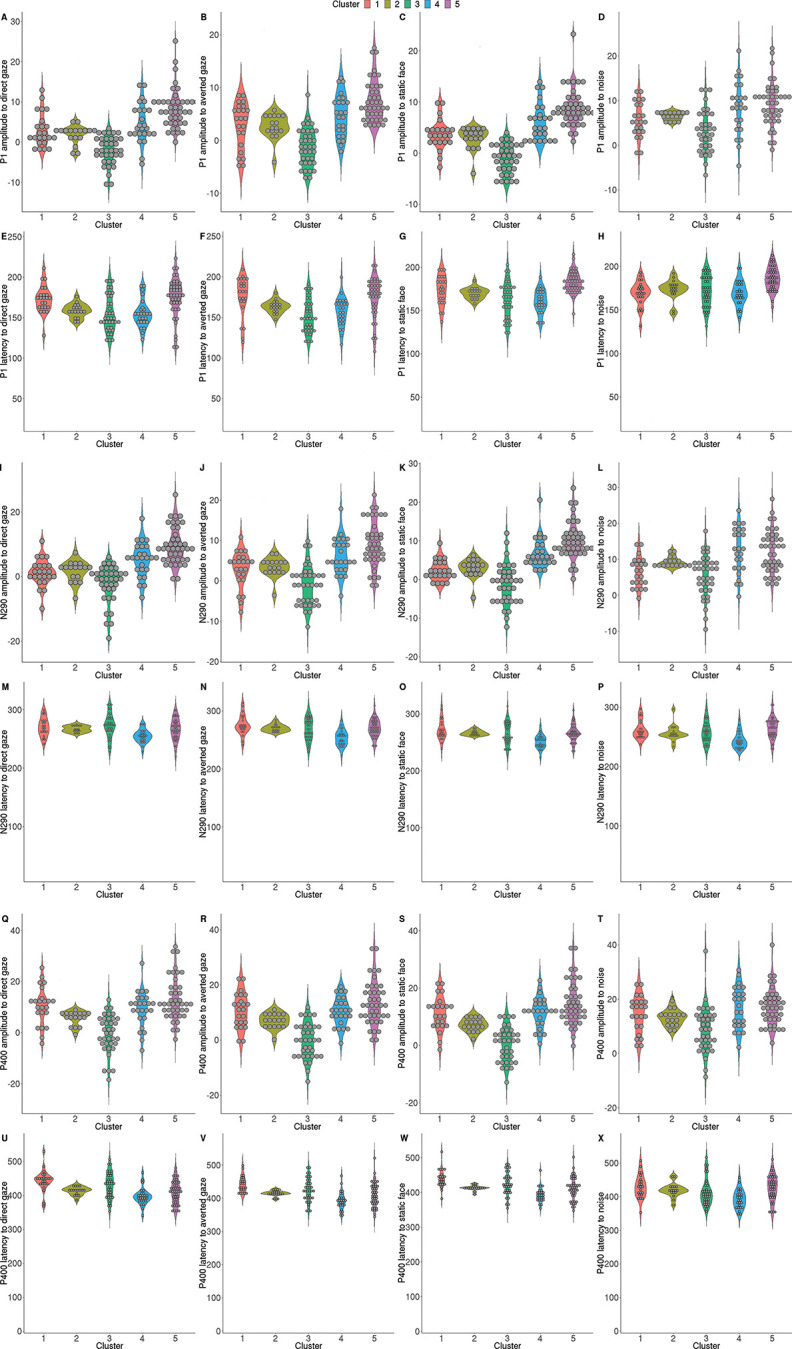
Subgroups of EL Infants Based on Face Processing in Infancy *Note*. Five clusters were identified among infants at elevated likelihood for ASD. This figure illustrates clusters comparisons on ERPs. Specifically, it shows only differences that were statistically significant after using Holm-Bonferroni correction for multiple comparisons: P1, N290, and P400 measured in amplitude and latency in response to direct gaze, averted gaze, static faces, and visual noise. See the online article for the color version of this figure.

We identified five stable clusters among EL infants indicating a diffuse pattern of differences in ERP components between subgroups for all stimuli except for dynamic gaze shifts. Cluster 1 (*n* = 23, 3 EL-ASD) showed slower P400 to faces and reduced N290 amplitude to visual noise; cluster 2 (*n* = 17, 4 EL-ASD) showed no specific differences compared to other clusters; cluster 3 (*n* = 37, 12 EL-ASD) showed reduced P1, N290, and P400 amplitude to faces and visual noise, and longer P400 latency to eye gaze; cluster 4 (*n* = 25, 2 EL-ASD) showed increased N290 amplitude and shorter P400 latency to visual noise, and shorter N290 latency to faces and visual noise; cluster 5 (*n* = 42, 11 EL-ASD) showed increased P1 and N290 amplitude, and longer P1 latency to faces and visual noise (see Figure 5, Table S7).[Fig fig5]

The identified clusters did not clearly map into clinical categories, and there were no significant differences between clusters on ASD symptoms, cognitive development, and adaptive functioning at 36 months (see Table S7). However, the majority of infants in the EL-ASD group were in clusters 3 and 5, which respectively suggests hypo- and hypersensitivity to faces and visual noise. To test this hypothesis, we compared clusters on sensory sensitivity total scores from the Infant-Toddler Sensory Profile (Dunn & Daniels, 2002) at 36 months, reflecting low neurological threshold to stimuli and passive self-regulation. We found a significant main effect of clusters through ANOVA, *F*(4, 139) = 3.99, *p* = .004; however, post hoc Tukey’s tests showed higher sensory sensitivity in infants from cluster 3 (*mean* [*standard deviation*] = 38.36 [11.48]) compared to cluster 4 (45.67 [8.92], *p* = .006) and cluster 5 (44.06 [8.75], *p* = .039).

## Discussion

We present a set of analytic approaches to investigate whether and how a particular RDoC-defined domain is linked to later psychopathology in the context of a prospective design. Specifically, we focused on face processing as a putative precursor of the social features emerging later in development that are characteristic of ASD. Findings indicate a diffuse pattern of alterations in both early sensory and later higher-level stages of neural processing of social (face/gaze) and visual noise stimuli in infancy that strongly converged to predict ASD diagnosis and social traits in toddlerhood across categorical and dimensional analyses (Table 1). These findings support a theoretical framework in which diffuse and individually heterogeneous anomalies in social and perceptual processing converge to contribute to the later emergence of autism.

### Neural Processing of Faces Is Diffusely Atypical in ASD

Our analyses converged to support the contention that the socially relevant processes of detecting a face and a shift in gaze are altered in emerging ASD across a long time-course of information processing from the shortest latency components, and across multiple stimuli conditions (Table 1). Our data-driven analyses identified a specific pattern of interactions between these different ERP components, rather than specific components, to be predictive of individual-level outcome. This supports hypothesis (a) on diversity of altered face processing to reflect consistent differences in different specific aspects of face processing that are masked in previous studies by their theory-driven approach on one or two components.

Prediction of dimensional (social functioning) and categorical outcome (ASD diagnosis) strongly converged in terms of patterns of neural responses to face/noise stimuli that were predictive of later ASD (Table 1). This contributes to validate the hypothesis of altered face processing as a candidate neurocognitive process underlying the emergence of ASD over development. Although categorical variables are less compelling to validate underlying mechanisms for psychopathology from an RDoC perspective, a diagnostic outcome retains a high clinical interest for decision-making in a medical system (Casey et al., 2013), and thus provided an opportunity to explore predictive power of altered face processing for ASD at different, complementary levels of analysis. In particular, both data-driven approaches identified the exact same responses to the face–noise contrast to be most predictive of later ASD, indicating an effect of social content of the stimulus on early alterations of face processing in ASD and a robust association to autism diagnosis and social traits in toddlerhood. Furthermore, neural responses to visual noise were selected among the most predictive features for socialization skills and ASD diagnosis in toddlerhood, suggesting that while alterations across different stages of face processing are the most salient features in emerging ASD, alterations in neural responses to nonsocial stimuli may also play a role. This is consistent with previous evidence of altered response to both social and nonsocial stimuli in emerging ASD (E. J. Jones et al., 2014), and extends recent work using nonlinear features of EEG signals to support widespread dysfunction not specific to social processing (Bosl et al., 2018).

Some components appeared to be more relevant to understanding the mechanisms underlying emergence of ASD social symptoms based on pervasiveness of effects across analyses. Specifically, N290 latency to visual noise versus faces emerged as a relevant feature across all levels of analysis (see Table 1), with infants in the EL-ASD group showing a diminished effect of longer N290 latency to faces compared to visual noise compared to TL and EL-no-ASD groups. N290 latency response to the face–noise contrast was selected as part of the “optimal” and “highest incidence” feature sets by the genetic algorithm, providing the best classification accuracies for ASD versus no-ASD infants (approximately 77% accuracy), and it had the largest negative correlation to dimensional outcome, with faster N290 response to noise compared to faces associated with better social functioning in toddlerhood. The reduced N290 latency difference between the social versus nonsocial stimuli in EL-ASD is in line with previous studies of EL siblings (E. Jones et al., 2016), and in the comparison of familiar and unfamiliar faces (Dawson et al., 2002; Key & Stone, 2012). The N290/P400 complex is thought to be a developmental precursor to the N170 (de Haan et al., 2003), an established marker for social functioning (Neuhaus et al., 2016) with a long history of research for alterations in ASD (Kang et al., 2018; McPartland et al., 2004). Early stage differences in neural processing, as we found here, may subsequently trigger a cascade of events that result in symptoms characteristic of ASD (Johnson et al., 2015). Reduced depth of processing for social stimuli may result, in fact, in failure to develop expertise in processing faces along a cumulative risk pathway.

Longer P1 latency to gaze shifting toward versus away from the infant could predict later ASD diagnosis at a group level, and could predict reduced social functioning in toddlerhood at an individual level. Similarly, longer P400 latency to gaze shifting toward versus away from the infant could predict later ASD diagnosis at a group level, and was included in the optimal feature set predicting individual ASD diagnosis with approximately 77% accuracy among siblings at elevated likelihood for ASD. Previous work on a subset of this cohort showed that reduced P100 latency differentiation of dynamic gaze predicts ASD outcome in toddlerhood (Elsabbagh et al., 2012), while reduced P400 differentiation of dynamic gaze predicts ASD outcome in middle childhood (Bedford et al., 2017). Here, neural responses to dynamic gaze shifts alone performed as best candidate precursors of ASD at a group level but did not provide sufficient predictive value at an individual level due to a significant overlap in individual variation. Nevertheless, neural responses to dynamic gaze were selected among relevant features for prediction of later ASD diagnosis and social traits, suggesting that alterations in neural processing of dynamic gaze are not sufficient but necessary conditions for emerging ASD.

### Stratification Into Subgroups of Face Processing

We identified five different subgroups of EL-infants differing in intensity and latency of response in early sensory (P1) and later higher-order stages (N290 and P400) of processing faces with direct and averted gaze and visual noise.

Infants with later ASD outcome were mainly partitioned into two subgroups characterized by lower (cluster 3) or higher (cluster 5) amplitude of neural responses to faces and visual noise, which was associated with respectively increased and reduced sensory sensitivity at 36 months. A previous study has found sensory sensitivity at age 2 years to predict greater attention capture by faces and more optimal social behavior at 4 years (E. Jones, Dawson, & Webb, 2018). Here, we looked at a younger age and found that infants who are less responsive to face–noise stimuli are more passive but also more sensitive responders to environmental stimuli in toddlerhood. In line with the idea of ASD as the result of early life adaptation to altered neural processing (Johnson et al., 2015), increased sensory sensitivity may result from a system that was initially underreactive but then amplifies sensory input over development. This could also explain why relations between neural responses to faces and sensory sensitivity seem to change over development, and highlights the need to investigate mechanisms across RDoC domains. Specifically, future studies should investigate the mechanisms underlying the association between neural sensitivity to social stimuli and sensory alterations observed in ASD. Nevertheless, sensory sensitivity may index a child’s likelihood to benefit from supportive environments during development. Thus, our findings indicate subgroups among infants developing ASD that could benefit most from different tailored intervention programs based on their sensory response to the environment.

Of note, differences between clusters in clinical outcome variables were not significant. While statistical power might improve with increased sample size, this can be interpreted in light of a conceptualization of ASD as an epiphenomenon of earlier-interacting susceptibilities (Constantino, 2018). Although altered face processing represents one of these early measurable liabilities to ASD, it might not fully capture alone the heterogeneity in clinical expression of the disorder. Future work should integrate longitudinal measures to further refine our understanding of subgroups of face processing in emerging ASD. Furthermore, future work might employ a different data-driven approach to parse individual heterogeneity and identify more homogeneous and replicable subgroups, which would potentially improve predictive accuracy and allow the identification of more specific physiological mechanisms (Bussu et al., 2019; Loth et al., 2017).

### Limitations and Future Directions

Early intervention for infants at elevated likelihood for ASD, prior to the emergence of core ASD features, is supported and conducted at the group level (Green et al., 2015; Green et al., 2017; Webb et al., 2014). Yet individual prediction of ASD in the first year of life might be crucial to enable targeted intervention within a critical developmental window. ERPs represent a cost-effective, mobile, and infant-friendly neuroimaging technology, providing potential utility for inclusion as proxy outcome markers for intervention trials. Future work should determine whether these parameters are sensitive to the effects of early intervention (E. J. H. Jones et al., 2017). It is important to consider methodological limitations in machine learning related to relatively small sample size and model reliability (Yahata et al., 2017). Currently, there is no good theoretical justification for features selected, and results may vary accordingly. Although we validated our results on a separate, holdout sample, generalizability of the identified model must be tested through replication on an independent sample. Furthermore, machine-learning algorithms were significantly but not strongly predictive of dimensional outcome, thus future work should explore incorporation of other measures (e.g., genetic factors, brain imaging, parent–child interaction, and behavioral measures) to capture multidimensional profiles.

### Conclusion

Our study represents the first attempt to investigate robustness and generalizability of findings on a specific RDoC domain (social cognition/face processing) in infancy across different levels of analysis, from group-based comparisons to individual-level prediction of outcome and stratification of ASD diagnosis and traits in toddlerhood, addressing the emerging need in developmental neuroscience to incorporate RDoC constructs. Prediction of categorical and dimensional outcomes strongly converge on a diffuse pattern of alterations across the time-course of neural processing of face/noise stimuli. Furthermore, we identified subgroups of infants based on neural sensitivity to faces in infancy that also show different sensory sensitivity in toddlerhood, which might guide tailoring interventions early in development to improve later outcome. This adds to the literature illustrating early structural alterations (Hazlett et al., 2017) by showing early diffuse functional alterations, in line with the idea of ASD as a syndrome emerging from diffuse and interacting liabilities (Constantino, 2018).

## Supplementary Material

10.1037/abn0000648.supp

## Figures and Tables

**Table 1 tbl1:** Summary of Findings Across Different Analyses

	Static gaze condition				Dynamic gaze shift condition				Face vs. noise condition		
Analysis	Direct gaze	Averted gaze	Direct vs. averted		Shifts towards	Shift away	Towards vs. away		Static face	Visual noise	Face vs. noise
Group-based comparison							P1 latency* P400 amplitude** P400 latency***				N290 latency****
Supervised classification^†^	P1 amplitude^#^ **N290 amplitude**^#^ **P400 amplitude**	**P1 amplitude** N290 amplitude **P400 amplitude**¥	P1 amplitude^#^ P400 latency^#^		N290 amplitude^¥^ **P400 amplitude**^#^	P400 amplitude^#^	P1 amplitude^#^ **P400 latency**^#^		P1 amplitude^#^ **N290 amplitude** **P400 amplitude**#	P1 amplitude^#^ P400 latency^#^	**P1 amplitude** **P1 latency****#** ***N290 latency*** **P400 amplitude**^**#**^
Prediction of dimensional outcome	P1 latency **N290 amplitude** N290 latency **P400 amplitude**	**P1 amplitude** P1 latency N290 latency **P400 amplitude**	N290 amplitude P400 amplitude		P1 amplitude **P400 amplitude** P400 latency	P1 amplitude P1 latency	**P1 latency** N290 amplitude		N290 latency **P400 amplitude** P400 latency	P1 latency	**P1 amplitude** **P1 latency** ***N290 latency*** **P400 amplitude**
Stratification into subgroups	P1 amplitude P1 latency N290 amplitude N290 latency P400 amplitude P400 latency	P1 amplitude P1 latency N290 amplitude N290 latency P400 amplitude P400 latency							P1 amplitude P1 latency N290 amplitude ***N290 latency*** P400 amplitude P400 latency	P1 amplitude P1 latency N290 amplitude ***N290 latency*** P400 amplitude P400 latency	
*Note*. This table shows the ERP measures that emerged as significant across the different analyses, in terms of significant differences between groups and/or being selected by the genetic algorithm for classification of EL-ASD vs. EL-no ASD and/or being selected by the elastic-net regression for prediction of later social skills and/or showing significant differences between subgroups of the EL group. Shaded gray indicates no significant results for the specific stimuli/conditions in a specific analysis. The measures appearing as significant at group and individual levels of analysis are highlighted in bold; significant at all levels of analysis in bold red (bold italic); and bold underlined when significant for prediction of both diagnosis and traits.											
* *F*(2, 213) = 4.95, *p* = .008; *shifts towards > shifts away* in TL (d = .74; *p* = .002) and EL-no-ASD (d = .43; *p* = .047). ** *F*(2, 212) = 4.13, *p* = .017; *shifts towards > shifts away* in EL-ASD (d = .46; *p* = .02) and EL-no-ASD (d = .41; *p* = .01). *** *F*(2, 208) = 3.51, *p* = .032; *shifts towards > shifts away* in TL (d = .55; *p* = .01) and EL-no-ASD (d = .47; *p* = .02); with *p* = .009 for EL-ASD vs. TL and *p* = .019 for EL-ASD vs. EL-no-ASD. **** *F*(2, 171) = 3.61, *p* = .029; *face > visual noise* in TL (d = .61; *p* = .01) and EL-no ASD (d = .52; *p* = .02). ^†^ The “optimal” feature set was the best performing classifier, with AUC = 77.1% [61.1, 90.5], *p* = .01; accuracy = 75.7% [69.1, 90.0]; sensitivity = 73.5% [41.2, 91.2]; specificity = 77.8% [66.7, 100]; PPV = 76.8% [71.2, 100]; NPV = 74.6% [62.7, 89.5]. ^#^ Features in the “optimal” but not in the “highest incidence” feature set. ^¥^ Features selected as part of the “highest incidence” but not the “optimal” feature set. See the online article for the color version of this table.
