# Correction to “Understanding the Nature of Face Processing in Early Autism: A Prospective Study” by Tye et al. (2022)

**DOI:** 10.1037/abn0001017

**Published:** 2025-08

In the article “Understanding the Nature of Face Processing in Early Autism: A Prospective Study,” by Charlotte Tye, Giorgia Bussu, Teodora Gliga, Mayada Elsabbagh, Greg Pasco, Kristinn Johnsen, Tony Charman, Emily J. H. Jones, Jan Buitelaar, Mark H. Johnson, and BASIS team (*Journal of Psychopathology and Clinical Science*, 2022, Vol. 131, No. 6, pp. 542–555, https://doi.org/10.1037/abn0000648), in the second paragraph of the Group-Level Differences section of the Results, in the sentence beginning “Specifically, the EL-ASD group had longer P1 latency to gaze shifting away versus toward …” the phrase “away versus toward” should have read “toward versus away.” In the same paragraph, “Finally, there was enhanced P400 amplitude to gaze shifting toward versus away in the EL-ASD group (*p* = .016, *d* = 0.46) and EL-no ASD group (*p* = .014, *d* = 0.41), with no difference between EL groups (*p* = .482, *d* = 0.12) and an opposite effect in the TL group” should have read “Finally, the TL group showed enhanced P400 amplitude to gaze shifting away versus toward the viewer, compared with reduced differentiation in both the EL-no ASD (*p* = .042, *d* = 0.41) and the EL-ASD (*p* = .046, *d* = 0.46) groups. There was no difference between the EL-no ASD and EL-ASD groups (*p* = .861, *d* = 0.12).”

In Panels A, B, and C of Figure 2, the TL and EL-no ASD groups are incorrectly keyed to each other’s colors. That is, green should represent the TL group and blue should represent the EL-no ASD group. In Panels A and B, the symbols for the conditions are also reversed. In Panel A, the circles should represent averted gaze and the diamonds should represent direct gaze. In Panel B, the triangles should represent gaze shift away and the squares should represent gaze shift toward. The corrected figure is below: 
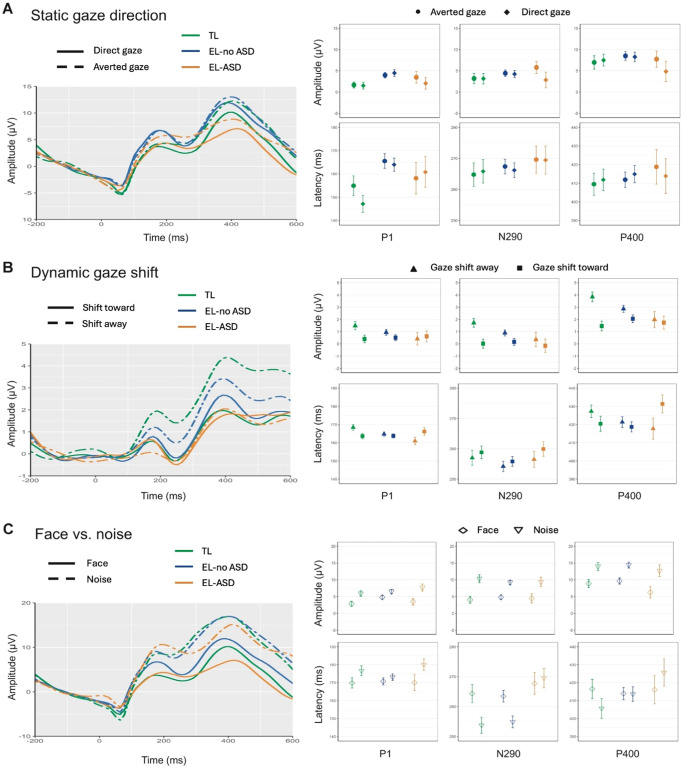


*Note.* See the online correction notice for the color version of this figure.

