# Supplementary material for: Understanding the Nature of Face Processing in Early Autism: A Prospective Study
Source: J Psychopathol Clin Sci. 2022 Aug;131(6):542–55. doi: 10.1037/abn0000648 (PMC9330670; doi:10.1037/abn0000648)
Supplement: Supplementary file 1 [file ABN-2020-1537_Suppl.docx]

**Understanding the nature of face processing in early autism: A prospective study.**

**Supplementary material**

Charlotte Tye^†^, Giorgia Bussu^†^, Teodora Gliga, Mayada Elsabbagh, Greg Pasco, Kristinn Johnsen, Tony Charman, Emily J.H. Jones^#^, Jan Buitelaar^#^, Mark H. Johnson^#^ and the BASIS team

^†^ shared first authorship

^#^ shared last authorship

# **Contents**

**Table S1**: Demographic and clinical characteristics of participants across EL and TL subgroups

**Table S2:** Demographic and clinical characteristics by cohort

**Table S3**: Valid trial numbers for participants included in ERP analysis by outcome group and by phase

**Table S4:** Performance metrics for prediction of 36-months ASD diagnosis

**Table S5:** Demographic and clinical characteristics of subgroups in EL infants

**Method S1:** Task

**Method S2:** Electrophysiological recording and analysis

**Method S3:** Genetic algorithm for feature selection

**Method S4:** Bayesian Hierarchical Clustering

**Analysis S1:** Association between group-level findings on P1 and P400 latency

**Analysis S2**: Cohort 2-only analysis

**Figure S1**: Selected channel montages

| ***Table S1: Demographic and clinical characteristics of participants across EL and TL subgroups*** | | | | | | |
| --- | --- | --- | --- | --- | --- | --- |
|  | **High-risk** | | | | **Low-risk** | **Group differences (TL, EL-no ASD, EL-ASD)** |
|  | *All* | *ASD* | *No ASD* | |  |  |
| **ERP at 8 months** | | | | | | |
| Age in days (SD) | 263.12 (34.31)  *n*=147 | 257.91 (35.54)  *n*=32 | 264.57 (33.98)  *n*=115 | | 257.91 (35.54)  *n*=68 | nsd |
| MSEL ELC | 102.17 (15.85)  *n*=147 | 97.37 (17.65)  *n*=32 | 103.50 (15.13)  *n*=115 | | 106.49 (12.35)  *n*=68 | p=.017; EL-ASD<TL |
| N (% boys) | 148 (49%) | 33 (76%) | 115 (42%) | | 68 (44%) | x^2^=12.40, p=.002 |
|  | | | | | | |
| **Outcome at 3 years** | | | | | | |
| Age in months (SD) | 38.47 (2.26)  *n*=146^c^ | 38.22 (1.93)  *n*=32 | 38.54 (2.35)  *n*=114 | 38.23 (2.29)  *n*=64 | | nsd |
| MSEL ELC (SD) | 103.12 (24.07)  *n*=145 | 87.94 (26.79)  *n*=31 | 107.25 (21.62)  *n*=114 | 116.81 (15.41)  *n*=64 | | p<.001; EL-ASD<EL-no ASD<TL |
| ADOS-2 social affect CSS (SD)^d^ | 3.53 (2.62)  *n*=146 | 5.03 (3.00)  *n*=32 | 3.11 (2.36)  *n*=114 | 3.22 (2.04)  *n*=64 | | p<.001; EL-ASD>EL-no ASD, TL |
| ADOS-2 restricted/repetitive behaviours CSS (SD)^d^ | 4.58 (2.70) | 6.56 (1.92) | 4.03 (2.63) | 3.72 (2.48) | | p<.001; EL-ASD>EL-no ASD, TL |
| ADOS-2 total CSS (SD)^d^ | 3.18 (2.60) | 5.09 (3.12) | 2.65 (2.16) | 2.56 (1.78) | | p<.001; EL-ASD>EL-no ASD, TL |
| ADOS-2 total raw score (SD)^d^ | 6.49 (5.50) | 11.03 (6.73) | 5.21 (4.34) | 5.02 (3.23) | | p<.001; EL-ASD>EL-no ASD, TL |
| ADI-R Social | 4.12 (5.17)  *n*=145 | 11.16 (5.64)  *n*=31 | 2.20 (2.88)  *n*=114 | - | | p<.001^e^; EL-ASD>EL-no ASD |
| ADI-R Communication | 4.28 (4.86) | 10.03 (5.15) | 2.72 (3.39) | - | | p<.001^e^; EL-ASD>EL-no ASD |
| ADI-R Behaviours/Repetitive Interests | 1.59 (2.37) | 4.61 (2.60) | 0.76 (1.46) | - | | p<.001^e^; EL-ASD>EL-no ASD |

^a^ includes participants in intervention case-series (see *Table S3*)

^b^ only includes participants in randomised controlled trial

^c^1 EL-ASD participant had incomplete MSEL

^d^ item scores from the ADOS-G were used to calculate ADOS-2 totals

^e^ADI-R not administered to TL group in cohort 1; indicates statistical tests between EL groups; nsd=non-significant difference.

Abbreviations: ADI-R: Autism Diagnostic Interview-revised; ADOS – Autism Diagnostic Observation Schedule; CSS = ADOS-2 Calibrated Severity Scale; MSEL ELC = Mullen Scales for Early Learning Early Learning Composite

***Table S2: Demographic and clinical characteristics by cohort (participants included in analyses)***

|  | **Cohort 1 (n=94)** | **Cohort 2 (n=122)** | **Group difference** |
| --- | --- | --- | --- |
| Male n (%) | 42 (40.38%) | 78 (54.54%) | x^2^ = 4.83, p=.028 |
| **ERP at 8 months** | | | |
| Age in days (SD) | 239.39 (37.66) | 276.53 (25.76) | t (155) = -8.17, p<.001 |
| MSEL ELC (SD) | 98.90 (12.95) | 107.07 (15.44) | t (213) = -4.11, p<.001 |
| **Outcome at 3 years** |  |  |  |
| Age in months (SD) | 37.87 (2.81) | 38.81 (1.62) | t (137) = -2.84, p=.005 |
| MSEL ELC (SD) | 110.16 (19.43) | 105.11 (24.71) | nsd |
| ADOS-2 social affect (SD) | 5.86 (3.87) | 3.69 (3.96) | t (208) = 3.97, p<.001 |
| ADOS-2 RRB (SD) | 1.47 (1.63) | 1.34 (1.50) | nsd |
| ADOS-2 CSS (SD) | 3.62 (2.44) | 2.51 (2.25) | t (208) = 3.41, p=.001 |
| ADI-R Social^a^ | 4.79 (5.45) | 3.78 (5.45) | nsd |
| ADI-R Communication^a^ | 4.56 (4.93) | 4.14 (4.85) | nsd |
| ADI-R Behaviours/Repetitive Interests^a^ | 1.60 (2.02) | 1.58 (2.53) | nsd |

Abbreviations: ADI-R: Autism Diagnostic Interview-revised; ADOS – Autism Diagnostic Observation Schedule; CSS = ADOS-2 Calibrated Severity Scale; MSEL ELC = Mullen Scales for Early Learning Early Learning Composite

^a^ADI-R not administered to TL group in cohort 1; indicates statistical tests between EL groups; nsd=non-significant difference.

***Table S3: Valid trial numbers for participants included in ERP analysis by outcome group and by phase***

| **Phase** | **Contrast** | | **TL** | **EL** | | |
| --- | --- | --- | --- | --- | --- | --- |
|  |  |  |  | **Combined** | **No ASD** | **ASD** |
| **Phase 1** | *Total sample* |  | n=50 | n=54 | n=37 | n=17 |
|  | *Static gaze* |  |  |  |  |  |
|  | Direct | Trials | 35.0 | 35.3 | 34.8 | 36.7 |
|  |  | Valid | 20.8 | 22.6 | 22.1 | 23.1 |
|  | Averted | Trials | 35.0 | 35.5 | 34.9 | 37.1 |
|  |  | Valid | 20.7 | 23.2 | 22.6 | 23.9 |
|  | *n* |  | *32* | *32* | *22* | *10* |
|  | *Gaze shift* |  |  |  |  |  |
|  | Toward | Trials | 128.6 | 127.5 | 129.1 | 123.6 |
|  |  | Valid | 58.7 | 63.0 | 63.6 | 59.2 |
|  | Away | Trials | 129.1 | 125.9 | 127.4 | 122.2 |
|  |  | Valid | 59.8 | 63.6 | 64.4 | 59.2 |
|  | *n* |  | *45* | *50* | *33* | *16* |
|  | *Face/noise* |  |  |  |  |  |
|  | Face | Trials | 69.0 | 67.1 | 66.5 | 68.4 |
|  |  | Valid | 39.4 | 39.4 | 39.4 | 37.9 |
|  | Noise | Trials | 46.8 | 45.9 | 45.3 | 47.9 |
|  |  | Valid | 26.5 | 26.7 | 27.2 | 25.5 |
|  | n |  | *35* | *41* | *27* | *13* |
|  |  |  |  |  |  |  |
| **Phase 2** | *Total sample* |  | n=27 | n=116 | n=99 | n=17 |
|  | *Static gaze* |  |  |  |  |  |
|  | Direct | Trials | 25.6 | 26.5 | 26.4 | 26.5 |
|  |  | Valid | 14.1 | 16.5 | 16.4 | 20.0 |
|  | Averted | Trials | 25.1 | 26.6 | 26.5 | 26.6 |
|  |  | Valid | 14.7 | 16.8 | 16.6 | 18.1 |
|  | n |  | *9* | *68* | *47* | *10* |
|  | *Gaze shift* |  |  |  |  |  |
|  | Toward | Trials | 99.7 | 105.4 | 105.1 | 105.7 |
|  |  | Valid | 44.5 | 51.1 | 49.8 | 56.9 |
|  | Away | Trials | 101.3 | 105.5 | 105.1 | 106.5 |
|  |  | Valid | 43.7 | 50.5 | 49.7 | 54.7 |
|  | *n* |  | *23* | *102* | *82* | *17* |
|  | *Face/noise* |  |  |  |  |  |
|  | Face | Trials | 50.7 | 53.1 | 52.9 | 53.1 |
|  |  | Valid | 24.1 | 28.0 |  | 32.6 |
|  | Noise | Trials | 37.5 | 39.3 | 39.2 | 38.9 |
|  |  | Valid | 19.3 | 22.2 | 21.7 | 24.8 |
|  | *n* |  | *20* | *84* | *67* | *12* |

| ***Table S4. Performance metrics for prediction of 36-months ASD diagnosis.*** | | | | | | | |
| --- | --- | --- | --- | --- | --- | --- | --- |
| **Classifier** | **AUC** | **p_chance_/p_optimal_** | **Accuracy** | **Sensitivity** | **Specificity** | **PPV** | **NPV** |
| All | 65.4  [51.0, 85.0] | 0.16/0.18 | 69.4  [62.4, 83.8] | 50.0  [33.3, 100] | 88.9  [33.3, 100] | 81.8  [60.0, 100] | 64.0  [60.0, 100] |
| Demographics | 69.6  [51.6, 93.1] | 0.16/0.19 | 77.8  [66.3, 94.4] | 55.6  [33.3, 100] | 100  [44.4, 100] | 100  [62.1, 100] | 69.2  [60.0, 100] |
| ERP contrasts | 67.6  [51.3, 88.2] | 0.02^*^/0.14 | 70.1  [63.1, 88.6] | 73.5  [29.4, 100] | 66.7  [33.3, 100] | 68.8  [59.3, 100] | 71.6  [58.6, 100] |
| Single ERPs | 56.2  [50.3, 74.8] | 0.34/0.03^*^ | 63.2  [60.5, 77.1] | 26.5  [23.5, 100] | 100  [26.5, 100] | 100  [56.9, 100] | 57.6  [56.7, 100] |
| Noise-face | 53.9  [50.3, 75.5] | 0.41/0.02^*^ | 62.4  [60.3, 80.9] | 47.1  [33.3, 100] | 77.8  [23.5, 100] | 67.9  [56.7, 100] | 59.5  [57.0, 100] |
| Static face | 65.7  [51.0, 84.7] | 0.33/0.03^*^ | 65.0  [60.3, 82.3] | 41.2  [32.3, 100] | 88.9  [22.2, 100] | 78.8  [56.3, 100] | 60.2  [57.6, 100] |
| Gaze shift | 56.9  [50.3, 77.5] | 0.22/0.03^*^ | 61.3  [59.2, 79.4] | 55.9  [29.4, 100] | 66.7  [29.4, 100] | 62.6  [56.8, 100] | 60.2  [56.8, 100] |
| **Optimal** | **77.1**  **[61.1, 90.5]** | **0.01^*^** | **75.7**  **[69.1, 90.0]** | **73.5**  **[41.2, 91.2]** | **77.8**  **[66.7, 100]** | **76.8**  **[71.2, 100]** | **74.6**  **[62.7, 89.5]** |
| Highest incidence | 77.5  [50.3, 74.8] | 0.02^*^/0.15 | 77.9  [60.5, 77.1] | 55.9  [23.5, 100] | 100  [26.5, 100] | 100  [56.9, 100] | 69.4  [56.7, 100] |
| Frequent noise-face | 73.5  [54.9, 90.2] | 0.34/0.05 | 73.0  [63.2, 88.6] | 66.7  [44.4, 100] | 79.4  [29.4, 97.1] | 76.4  [58.6, 95.8] | 70.4  [61.4, 100] |
| Frequent static face | 74.8  [53.9, 93.1] | 0.87/0.04^*^ | 78.9  [64.9, 94.1] | 66.7  [44.4, 100] | 91.2  [55.6, 100] | 88.3  [64.1, 100] | 73.2  [61.4, 100] |
| This table shows performance metrics of classifiers for different input sets of features discriminating EL sibling who developed ASD from those who did not (*EL- ASD vs EL- no ASD*). The significance difference of classification AUC from chance level and from the optimal classifier were determined by permutation tests, the resulting *p-values* are reported respectively as *p_chance_* and *p_optimal_*. Differences were considered significant if *p*<0.05 [marked as ^*^]. All measures are reported as percentage, with 95% bootstrap confidence interval (CI) is reported in parentheses. The best performing classifier is highlighted in bold red.  *Abbreviations:* AUC = area under the curve; PPV = positive predictive power; NPV = negative predictive power.  **All:** gender, age, P1/N290/P400 amplitude and latency in response to gaze shifts directed towards the infant (SD), to gaze shifts directed away from the infant (SA), to static face with direct gaze (FD), to static gaze with averted gaze (FA), to visual noise (N), to static face with either direct or averted gaze (F), and differential responses to the SD-SA, FD-FA and N-F contrasts.  **Demographics:** gender, age;  **ERP contrasts:** differential responses to the SD-SA, FD-FA and N-F contrasts measured by P1/N290/P400 amplitude and latency.  **Single ERPs:** P1/N290/P400 amplitude and latency in response to the SD, SA, FD, FA, N, F stimuli.  **Noise-face:** P1/N290/P400 amplitude and latency in response to the N and F stimuli.  **Static face:** P1/N290/P400 amplitude and latency in response to the FD and FA stimuli.  **Gaze shift:** P1/N290/P400 amplitude and latency in response to the SD and SA stimuli.  **Optimal:** gender, P1 amplitude and P400 latency in response to the SD-SA contrast; P1 amplitude and P400 latency in response to FD-FA contrast; P1 amplitude and latency, N290 latency and P400 amplitude in response to the N-F contrast; P400 amplitude in response to SD and SA; P1 amplitude and N290 amplitude in response to FD and FA; P400 amplitude in response to FD; P1 amplitude and P400 latency in response to N; P1/N290/P400 amplitude in response to F.  **Highest incidence:** P1 amplitude and N290 latency in response to the N-F contrast; N290 amplitude in response to SD; P1/N290/P400 amplitude in response to FA; P400 amplitude in response to FD; N290 amplitude in response to F.  **Frequent noise-face:** P1 amplitude and N290 latency in response to the N-F contrast.  **Frequent static face:** P1/N290/P400 amplitude in response to FA; P400 amplitude in response to FD; N290 amplitude in response to F. | | | | | | | |

***Table S5: Demographic and clinical characteristics of subgroups among EL infants***

|  | | *Cluster 1*  *(n=23)* | *Cluster 2*  *(n=17)* | | *Cluster 3*  *(n=37)* | | *Cluster 4*  *(n=25)* | *Cluster 5*  *(n=42)* |
| --- | --- | --- | --- | --- | --- | --- | --- | --- |
| Age in months (SD) | | 8.35  (0.65) | 8.24  (1.09) | | 7.81  (1.35) | | 8.64  (1.15) | 8.31  (1.05) |
| N (% boys)* | | 17 (74%) | 6 (35%) | | 19 (51%) | | 6 (24%) | 27 (64%) |
| MSEL [mean (SD)] | |  |  | |  | |  |  |
| FM | | 49.09  (18.73) | 46.29  (17.19) | | 45.0  (17.40) | | 55.50  (13.84) | 47.56  (16.41) |
| VR | | 56.61  (17.75) | 48.76  (19.0) | | 54.23  (14.58) | | 62.58  (10.43) | 52.56  (11.50) |
| RL | | 52.91  (14.98) | 45.41  (16.58) | | 46.67  (14.39) | | 54.83  (7.64) | 48.39  (11.91) |
| EL | | 54.57  (16.17) | 49.76  (16.13) | | 48.97  (13.35) | | 55.92  (8.40) | 49.71  (14.44) |
| VABS [mean (SD)] | |  |  | |  | |  |  |
| Comm | | 97.87  (15.19) | 96.82  (16.99) | | 91.82  (15.08) | | 102.8  (13.15) | 98.50  (12.62) |
| DL | | 103.3  (12.98) | 99.24  (14.66) | | 95.68  (15.67) | | 106.2  (12.91) | 100.6  (12.86) |
| Mot | | 88.43  (9.44) | 86.82  (10.88) | | 90.5  (15.03) | | 95.08  (11.55) | 90.69  (11.55) |
| Soc | | 95.09  (12.66) | 93.94  (12.15) | | 89.97  (15.44) | | 98.08  (11.78) | 94.74  (12.58) |
| ADOS-2 [mean (SD)] | |  |  | |  | |  |  |
| CSS-SA | | 3.26  (2.36) | 4.29  (3.02) | | 3.75  (2.80) | | 3.08  (2.48) | 3.36  (2.63) |
| CSS-RRB | | 5.48  (2.19) | 3.94  (3.09) | | 5.0  (2.65) | | 3.88  (2.35) | 4.45  (2.86) |
| CSS-Tot | | 3.09  (2.61) | 3.59  (3.14) | | 3.44  (2.71) | | 2.58  (2.28) | 3.19  (4.91) |
| ADI-R | |  |  | |  | |  |  |
| Soc | | 4.0  (5.46) | 3.82  (4.81) | | 5.03  (5.80) | | 2.56  (3.10) | 3.85  (4.91) |
| Comm | | 4.35  (4.51) | 3.82  (5.56) | | 5.03  (4.95) | | 2.76  (3.18) | 3.98  (4.67) |
| RBI | | 1.39  (2.15) | 1.0  (1.41) | | 1.68  (2.31) | | 1.20  (2.38) | 1.90  (2.74) |
| SCQ [mean (SD)] | |  |  | |  | |  |  |
| Tot | | 6.70  (8.64) | 6.24  (6.01) | | 8.03  (6.41) | | 4.60  (5.68) | 6.0  (6.62) |
| ITSP [mean (SD)] | |  |  | |  | |  |  |
| Sensory sensitivity** | | 42.84  (8.18) | 48.0  (5.50) | | 38.36  (11.48) | | 45.67  (8.92) | 44.06  (8.75) |
| * χ^2^(4)=16.73, p=0.002  ** F(4,139)=3.99, p=0.004. Post-hoc Tukey’s tests showed lower sensory sensitivity in infants from cluster 3 compared to cluster 4 (p=0.006) and cluster 5 (p=0.039).  *Abbreviations:* EL = elevated likelihood for ASD; MSEL = Mullen Scales for Early Learning; MSEL-FM = MSEL fine motor domain score; MSEL-VR = MSEL visual receptive domain score; MSEL-RL = MSEL receptive language domain score; MSEL-EL = MSEL expressive language domain score; VABS = Vineland Adaptive Behavior Scales; ADI-R: Autism Diagnostic Interview-revised; VABS-Comm = VABS communication domain score; VABS-DL = VABS daily living domain score; VABS-Mot = VABS motor domain score; VABS-Soc = VABS social domain score; ADOS – Autism Diagnostic Observation Schedule; CSS-SA = social affect subscale of the ADOS-2 Calibrated Severity Scale; CSS-RRB = restricted and repetitive behaviours subscale of the ADOS-2 Calibrated Severity Scale; CSS-Tot = ADOS-2 Calibrated Severity Scale total score; ADI-R Soc = ADI-R social score; ADI-R Comm = ADI-R communication score; ADI-R RBI = ADI-R restricted behaviours and interests score; SCQ-Tot = total score of the Social Communication Questionnaire; ITSP = Infant-Toddler Sensory Profile. | | | | | | | | |
|  |  | | |  | |  |  |  |

## **Supplementary methods**

**Method S1: Task**

Infants sat on their parents’ laps at a 60cm distance from a computer screen. Gaze was recorded by video camera. Each trial block began with a static colourful fixation stimulus followed by a colour image of one of four female faces, with gaze directed either toward or away from the infant. In subsequent trials of the same block, the face remained on the screen but displayed 3-6 gaze shifts, alternating from directed toward to away from the infant. Eyes appeared at the same location as the fixation stimuli, to ensure infants were fixating the eye region. In addition to face trials, during approximately one third of all blocks, infants were presented with “visual noise” stimuli, constructed from the same faces presented within the task by randomizing the phase spectra while keeping the amplitude and colour spectra constant. Fixation stimuli were presented for a variable duration of 800 to 1200ms. Each trial lasted for 800ms (full description in (Elsabbagh et al., 2012)).

**Method S2: Electrophysiological recording and analysis**

EEG was recorded from a 128 channel Hydrocel Sensor Net. The reference electrode was positioned at the vertex (Cz in the conventional 10/20 system). The signal was digitized at a 500Hz sampling rate and band-pass filtered between 0.1-100Hz. Data were stored and analysed offline in EGI Netstation version 5.2.0.2 (using the same protocol as (Elsabbagh et al., 2012)). Trials were retained only when infants were fixating on the centre of the screen at stimulus onset, without any gaze shifts, blinking or head movements during the 800ms segment following stimulus onset. Data were then corrected to the -200ms baseline. Following automated artifact detection, an experienced EEG researcher (CT) conducted detailed manual artifact rejection through visual inspection of individual trials. Data from any sensor were excluded if they contained artifacts. Missing data from 12 or fewer channels were interpolated, otherwise the entire trial was rejected. Data were then rereferenced to the average.

Stimulus-locked epochs (-200 to 800ms peristimulus window) were averaged for the following trial contrasts: (1) faces (valid static (irrespective of gaze direction) vs. visual noise stimuli presented at the beginning of each block); (2) static gaze (faces with direct vs. averted gaze presented at the beginning of each block); and (3) dynamic gaze shifts (gaze toward vs. away from the infant, after appearance of the initial face within each block). Averages were computed for each participant in each condition on a minimum of 10 trials. Due to variable rates of presentation of each stimulus type, a different number of trials were included for each contrast, which did not differ by outcome group (*Table S3*). The occipito-temporal montages from Elsabbagh et al. (2012) were used (*Figure S1*) and corroborated with visual inspection of the grand average for each condition across the three contrasts. Peak amplitude and latency of the average P1, N290 and P400 responses were included in subsequent analyses because consistently modulated in face processing tasks in the first year of life.

### **Method S3: Genetic Algorithm for Feature Selection**

Feature selection is the process of finding the most relevant variables for the predictive model to reduce redundancy in the set of variables. Redundancy might in fact degrade accuracy, generalization and learning speed of the model (Dash, 1997). The genetic algorithm (Johannesson et al., 2002) is one of the most advanced algorithms for feature selection. It is a stochastic method for function optimization inspired by the evolutionary process of natural selection on genotype which inspired the algorithm, but it does not necessarily involve genetic data and can be applied to any kind of features. Starting from a collection (population) of candidate solutions (chromosomes; here sets of features) built from the available measures (gene pool; here features), the evolutionary process begins generating successive populations (generations) through mating, crossover and mutation (Back, 1996). The fitness is computed for each chromosome in each generation, and selection is based on the Darwinian principle of survival of the fittest, which in the end provides the best solution for the search problem. For reproduction, chromosomes are selected by evaluating the fitness value. Chromosomes with higher fitness have higher chance to be elected into the recombination pool.

In the present study, fitness is a measure of predictive performance of a 10-fold cross-validated SVM classifier built on the set of features under evaluation [chromosome]. We chose the Area Under the Curve (AUC) as the target value for fitness. The AUC is a measure of predictive accuracy for the model, computed as the area under the Receiver Operating Characteristic (ROC) curve, where the ROC curve is a plot of true positive rate vs. false positive rate for the model under evaluation.

Population size [n=100] and number of generations [n=200] were selected by an experienced researcher (KJ). The number of features was selected based on the AUC level reached during the evolutionary process, and stability of the process assessed through visual inspection. Once selected the number of features (n=21), the evolutionary process was repeated n=100 times to investigate the variability in the feature space. The feature set providing the highest AUC was selected as input for the subsequent classifier analysis (optimal set). In addition to it, feature sets with highest AUC (higher than 85%) were used for frequency analysis on the selected features. In fact, these candidate solutions have nearly equal quality for classification, but the incidence frequency of each feature in the genetic evolutionary process provides an estimate of the relevance of each feature for the specific classification problem. The features with highest incidence (higher than 80%) were selected as input for subsequent classifier analysis (highest incidence set), as well as task-based subsets of this highest incidence set.

### **Method S4: Bayesian Hierarchical Clustering**

Bayesian Hierarchical Clustering (BHC) is a model-based clustering algorithm built on a Dirichlet process mixture to model uncertainty in the data. Compared to other clustering techniques, it overcomes common limitations such as relatively arbitrary selection of number of clusters, or distance metric (Marrelec, Messe, & Bellec, 2015). It uses, in fact, marginal likelihoods to decide which clusters to merge at each step of a bottom-up hierarchical clustering process. The use of Bayesian hypothesis-testing as model-based criterion for merging clusters is also advantageous in terms of quality of the resulting clusters compared to the use of ad-hoc distance metrics.

## **Supplementary analysis**

### ***Analysis S1: Association between group-level findings on P1 and P400 latency***

No significant associations between latency of the P1 and P400 ERP difference scores for gaze shift towards versus away were found (whole sample: r=0.3, p=.697; TL: r=-.05, p=.648; EL-no ASD: r=.05, p=.622; EL-ASD: r=-.08, p=.664). A hierarchical regression indicated independent effects of the P1 and P400 difference scores on outcome group; the P1 latency difference remained after entering the P400 difference (beta=.211, p=.002) and the P400 latency difference remained after entering the P1 difference (beta=.160, p=.017). In combination with the effect of age and non-verbal ability specifically on P1 latency, this suggests that the P400 difference is not directly attributable to inputs from earlier stage processing. Rather, early stages of responses might index something different, like hypersensitivity in relation to social attention (Jones, Dawson, & Webb, 2018).

### ***Analysis S2: Cohort 2-only analysis***

**Face/noise contrast.**

A significant condition x outcome interaction emerged on N290 latency (F (2, 96) = 4.80, p=.010). The HR-ASD group showed a diminished effect of face versus noise compared to the LR (p=.003, d=1.12) and HR-no ASD (p=.016, d=0.71) groups, with no difference between LR and HR-no ASD (p=.162, d=0.38). There were no other significant interactions (all ps>.05).

**Dynamic gaze contrast.**

There was a significant condition x outcome interaction on P400 latency (F (2,114)=3.55, p=.03), whereby the LR and HR-no ASD groups showed longer latency to gaze shifting away versus towards, compared to HR-ASD (LR: p=.010, d=0.92; HR-no ASD: p=.045, d=0.53). There was no significant difference between LR and HR-no ASD (p=.187, d=0.35). No other interactions were significant (all ps>.05).

**Static gaze contrast.**

There was a significant condition x outcome interaction on P400 latency (F (2,64)=3.55, p=.035), whereby the LR group showed a longer latency to direct static gaze compared to averted gaze, compared to the HR=no ASD (p=.021, d=0.88) and the HR-ASD groups (p=.015, d=1.40). There was no significant difference between the HR groups (p=.042, d=0.31). There were no other significant interactions for the static gaze contrast (all ps>.05).

**Figure S1**: Selected channel montages based on Elsabbagh et al. (2012) and corroborated with visual inspection of grand averages


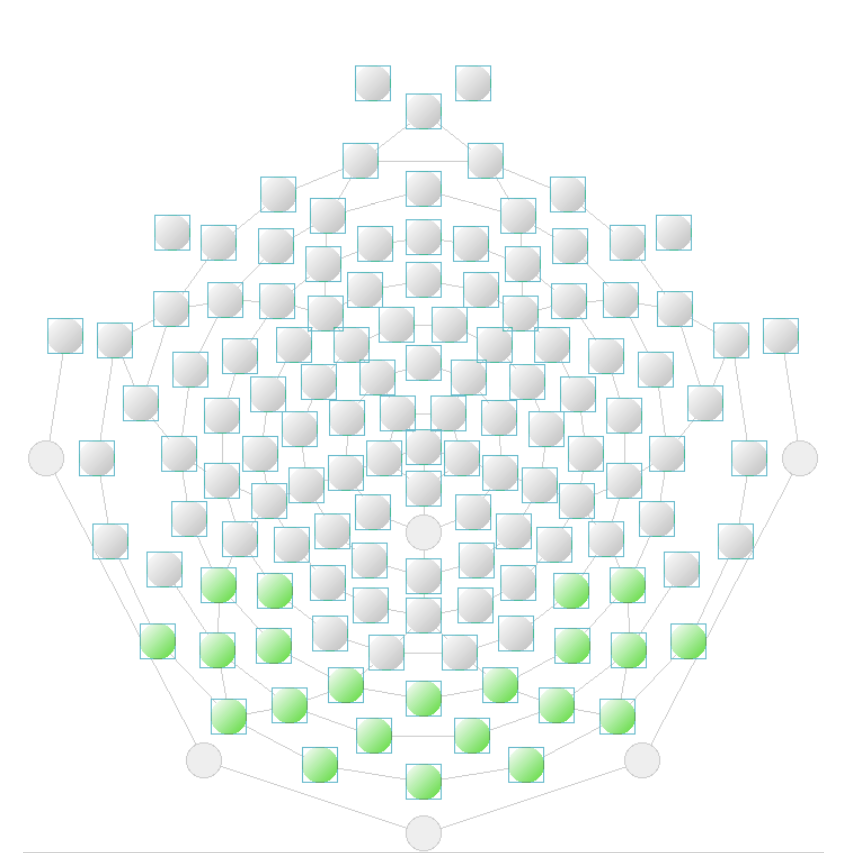


Static gaze contrast montage


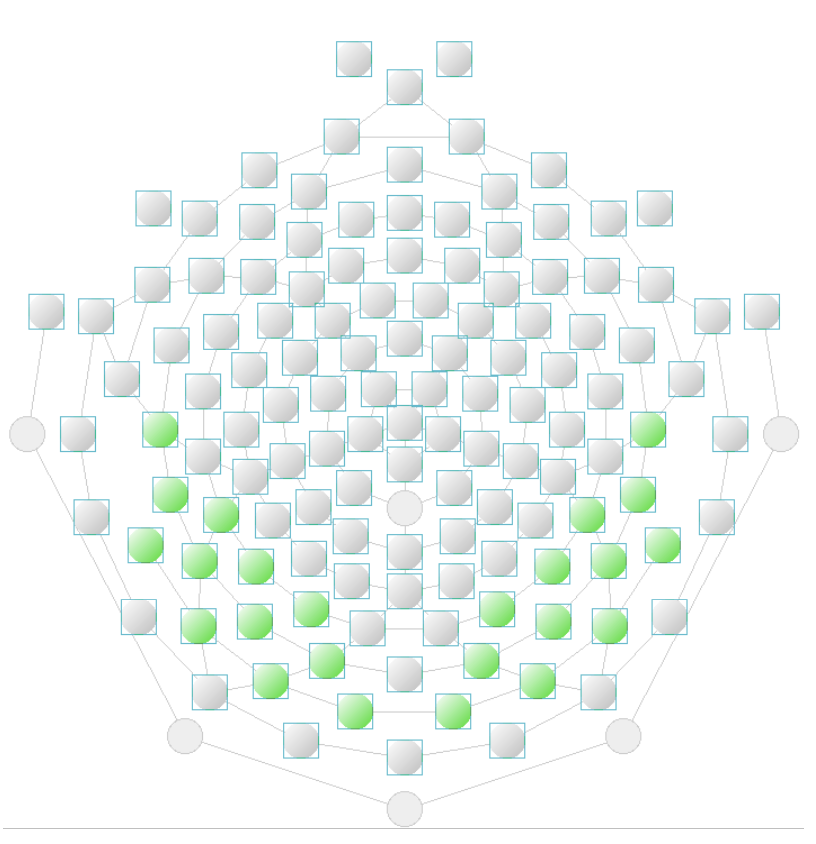


Dynamic gaze contrast


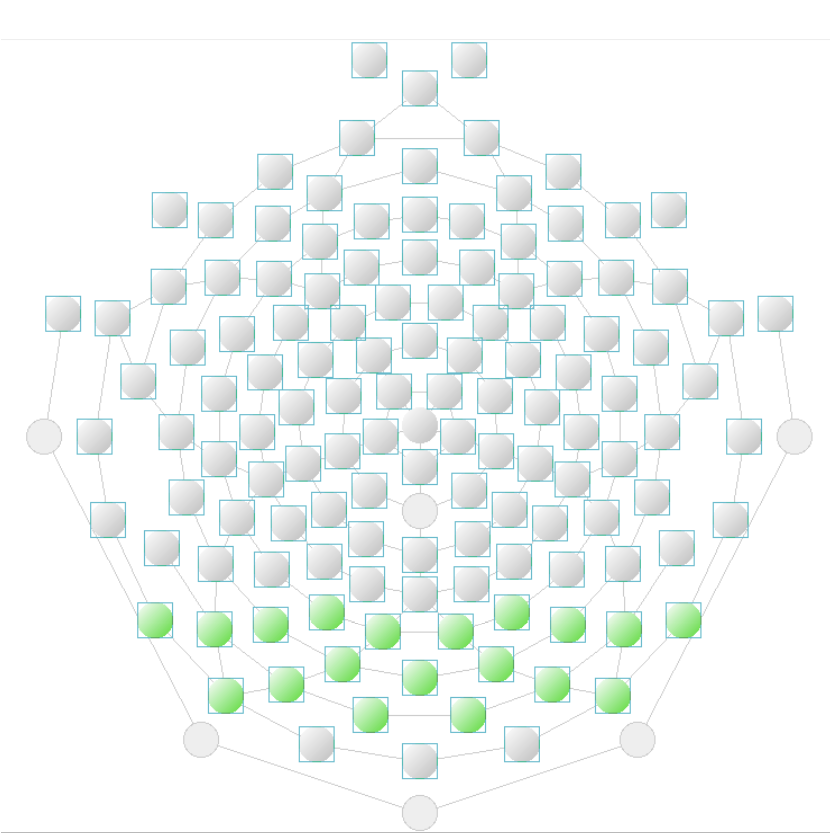


Face/noise contrast

References

Back, T. (1996). Evolution strategies: An alternative evolutionary algorithm. *Artificial Evolution, 1063*, 3-20.

Dash, M. a. L., H. (1997). Feature selection for classification. *Intelligent Data Analysis, 1*, 131-156.

Elsabbagh, M., Mercure, E., Hudry, K., Chandler, S., Pasco, G., Charman, T., . . . , B. A. S. I. S. T. (2012). Infant neural sensitivity to dynamic eye gaze is associated with later emerging autism. *Curr Biol, 22*(4), 338-342.

Johannesson, G. H., Bligaard, T., Ruban, A. V., Skriver, H. L., Jacobsen, K. W., & Norskov, J. K. (2002). Combined electronic structure and evolutionary search approach to materials design. *Phys Rev Lett, 88*(25 Pt 1), 255506. doi:10.1103/PhysRevLett.88.255506

Jones, E., Dawson, G., & Webb, S. (2018). Sensory hypersensitivity predicts enhanced attention capture by faces in the early development of ASD. *Developmental cognitive neuroscience, 29*, 11-20.

Marrelec, G., Messe, A., & Bellec, P. (2015). A Bayesian Alternative to Mutual Information for the Hierarchical Clustering of Dependent Random Variables. *PLoS One, 10*(9), e0137278. doi:10.1371/journal.pone.0137278
